# Generic Anticancer Drugs of the Jan Aushadhi Scheme in India and Their Branded Counterparts: The First Cost Comparison Study

**DOI:** 10.7759/cureus.19231

**Published:** 2021-11-03

**Authors:** Thomas George, Manjeshwar S Baliga

**Affiliations:** 1 Internal Medicine, Coney Island Hospital, Brooklyn, USA; 2 Research, Mangalore Institute of Oncology, Mangalore, IND

**Keywords:** cost variance percent, cost variance, anticancer drugs, indian market, jan aushadhi, pharmacoeconomic

## Abstract

Introduction: In India, the costs of cancer drugs are exorbitant and cause significant financial toxicity to the affected patient and their family members. Considering this, Jan Aushadhi pharmacy stores were established across the country by the government of India with the objective of providing cheap generic medicines to patients. The objective of this study was to perform a cost comparison study of generic chemotherapeutic drugs provided through Jan Aushadhi pharmacies versus their branded counterparts. This will help patients and physicians get first-hand information on the cost variation between generic and branded anticancer drugs in India.

Materials and Methods: The cost of Jan Aushadhi generic drugs and the cost of the most expensive and cheapest marketed branded drugs for the same molecule and dose were ascertained and presented in both Indian Rupees (INR) and US Dollars (as of July 2021). Finally, the difference in costs in INR, cost ratio, and cost variance were calculated, comparing the price of the Jan Aushadhi generic drugs with the most expensive and the cheapest branded drug in the same category.

Results: Compared to branded drugs, all the Jan Ausadhi generic drugs were cheaper, except one (methotrexate). The highest cost difference was observed for docetaxel, while the least was observed for methotrexate tablets (2.5 mg). The highest cost ratio (22.24) and cost variance (3327.56) were observed for doxorubicin injection (50 mg).

Conclusion: The current study compares the cost difference between the marketed branded and Jan Aushadhi generic anticancer drugs for the first time. Replacing the costly branded anticancer drugs with Jan Aushadhi generic drugs can result in substantial cost savings. The information obtained from this cost difference analysis will be helpful for the healthcare fraternity, patients, policymakers, and society at large.

## Introduction

Cancer is a major health issue in India. The International Agency for Research on Cancer’s GLOBOCAN project estimates that compared to 2012 where almost one million cases were reported, the burden will almost double in 2035 to a projected 1.7 million, and the number of cancer-related deaths will increase from 0.68 to 1.2 million [1]. The age-standardized prevalence of cancer is estimated to be 97 per 100,000 and causes 6% of all deaths [2]. Reports also indicate that gastric, breast, lung, lip, and oral cavity, pharynx other than nasopharynx, colon and rectum, leukemia, cervical, esophageal, brain, and nervous system were the leading cancers in India [3].

Globally, cancer treatment is very structured and clearly defined with proper guidelines to be strictly adhered to by the treating physician/s [4]. The proposed treatment strongly advocates judicious use of surgery, chemotherapy, and radiotherapy depending on the stage and general health of the individual and that the sequence of treatment modality to be adopted should be arrived at through detailed discussion in the tumor board consisting of experienced oncologists [5]. Clinically, when the tumor is localized, operable, and the patient's general health is good, surgery is the first choice of treatment [4]. Following surgery, radiotherapy (for example, lung, head, and neck cancer) or chemotherapy (like in ovarian, gastric, pancreatic, and colon cancer) and at times both (for example, breast) may be used to achieve optimal elimination of the residual neoplastic/mutated cells to increase the chances of complete cure [4].

When the patient’s general health is poor, and/or tumor is inoperable, as with a deep-seated brain tumor, loco-regional growths like in the oropharyngeal cancers, radiotherapy is the most preferred modality. It may be used either alone or combined with chemotherapy (chemo-irradiation) [6]. However, when the tumor is locally advanced or highly malignant and has spread to distant organs from the genesis site, chemotherapy is the most preferred treatment modality [4]. Chemotherapy is also used before surgery (neoadjuvant treatment) and with or without radiotherapy to reduce the size of the tumor, which is subsequently excised through surgery [4]. Also, it may be used to exterminate the residual neoplastic cells in the margins of the excised regions to enhance the cytotoxic effects of ionizing radiations and kill the neoplastic cells that may have undergone micrometastases from the primary site to adjacent or distant organs [4].

From an economic viewpoint, although beneficial, chemotherapy is expensive and incurs a huge financial burden on the patient and their family [7]. For most Indians, the cost of cancer treatment and the requisite medicines are colossal and amount to a substantial part of their lifelong savings [7]. Also, seminal studies by Goldstein and co-workers in 2017 have shown that although the drug prices were highest in the United States (calculated in US $), when compared from the cost of affordability, it was the least affordable in India [8]. This is primarily because more than 90% of Indians do not have health insurance, and out-of-pocket expenditure is a necessity in these people when a family member is affected by any serious health ailment like cancer [7,9].

Considering the cost burden of marketed branded drugs on marginalized populations, in the year 2008, the government of India launched the Jan Aushadhi scheme (a Hindi term that translates as “people’s medicine”), a public medicine scheme of making generic medicines available at affordable prices in select designated pharmacy stores termed as “Jan Aushadhi Kendra” [10,11]. Jan Aushadhi Kendra dispenses most commonly used drugs that do not need a license from the innovator company. The government of India regulates the cost of both scheduled and nonscheduled medicines [10,11]. Additionally, the drugs do not have excise duty. A uniform nominal value-added tax of 4% is applied to make drugs affordable to society's marginalized and impoverished people [12]. The Jan Aushadhi Kendra also dispenses chemotherapeutic drugs on a validated prescription of an oncologist.

In pharmacy research, cost-minimization analysis (CMA) is extremely important and is aimed at ascertaining the least costly drug or a combination to reduce the financial toxicity to the patient and society [13]. Previous studies by Kolasani and co-workers have shown that a substantial difference exists in the cost variance (CV) for different brands (marketed in the same dose and dosage) of the approved anticancer drugs in India [14]. With regard to the drugs available at the Jan Aushadhi stores, reports suggest that when compared to the branded drugs, a substantial variation in the costs exists for psychotropic medicines [15], anticancer drugs used for treating breast cancer [13], and for anticoagulants, antiplatelets, and fibrinolytic used for thromboembolic disorders [16]. For the first time, this study compares the cost of generic anticancer drugs available at Jan Aushadhi pharmacies with the costliest and cheapest branded drugs with the same molecular composition and dose marketed throughout India.

## Materials and methods

The cost of the anticancer drugs available at Jan Aushadhi Kendra was ascertained by referring to the current master list of the Pradhan Mantri Bharatiya Jan Aushadhi scheme brochure and from the Pharmaceuticals & Medical Devices Bureau of India [17]. As hundreds of companies manufacture or market drugs of similar formulations at varied costs, it was decided to note each drug's costliest and cheapest formulation. The costs of the most expensive and least expensive branded drug were noted from the Current Index of Medical Stores [18] and used for the study.

The cost for each unit or tablet or capsules or injections was considered in Indian Rupees (INR) and US Dollars (1 dollar = INR 74.39 on July 29, 2021). The cost difference (CD) was calculated by deducting the cost of Jan Aushadhi from that of the costliest and cheapest brand. Cost ratio was calculated using the formula: maximum cost ÷ minimum cost; while CV was calculated as (maximum cost - minimum cost) x 100 ÷ minimum cost. The data on variations in the price has been represented in actual cost per unit in INR, US Dollar, and percentages. Adhering to ethical concerns and fluctuating market prices, the brand name of each drug's mentioned costliest and cheapest formulation or their manufacturers is not disclosed.

## Results

A total of 27 drugs were considered of which 19 were cytotoxic drugs, five were drugs that modulated hormone levels or their action, two were immune modulators, and one was a bisphosphonate used as an adjunct to reduce bone loss essential in cancer care. The results on actual cost, CD, CR, and CV by comparing the Jan Aushadhi generic drugs with the costliest and cheapest branded drugs are represented in Table 1.

**Table 1:** Comparison of difference in actual costs, cost ratio, and cost variance between the Jan Aushadhi generic drugs and expensive and inexpensive branded anticancer drugs marketed in India. "Costliest" refers to the costliest available branded drug and "Cheapest" refers to the cheapest available branded drug. JAS: Jan Aushadhi scheme

Generic name (class of drug and mechanism of action)	Drug dose tab/strip or mg/ vial	Cost of single tablet/unit in INR (in US Dollars)			Difference in actual cost price in INR		Cost ratio		Cost variance	
		JAS	Costliest	Cheapest	Costliest	Cheapest	Costliest	Cheapest	Costliest	Cheapest
Cytotoxic drugs										
5-Fluorouracil (Antimetabolite drug)	Inj. 500 mg/ 10ml	11.50 (0.15)	114.87 (1.54)	20.77 (0.28)	103.37	9.27	9.99	1.81	898.87	80.61
Bleomycin (Antitumor antibiotics)	Inj. 15 mg vial	300.00 (4.03)	2855.00 (38.38)	681.63 (9.16)	2555.00	381.63	9.52	2.27	851.67	127.21
Capecitabine (Antimetabolite)	Tab. 500 mg 10's	42.00 (0.56)	159.90 (2.15)	112.711 (1.52)	117.90	70.71	3.81	2.68	280.71	168.36
Carboplatin (alkylating agent)	Inj. 150mg/ 15ml	375.00 (5.04)	2411.19 (32.41)	777.74 (10.45)	2036.19	402.74	6.43	2.07	542.98	107.40
	Inj. 450 mg/ 45ml	1707.00 (22.95)	4725.00 (63.52)	2333.21 (31.36)	3018.00	626.21	2.77	1.37	176.80	36.68
Cisplatin (alkylating agent)	Inj. 50 mg/ 50ml vial	156.00 (2.10)	350.00 (4.70)	278.57 (3.74)	194.00	122.57	2.24	1.79	124.36	78.57
	Inj. 10 mg/ vial	32.00 (0.43)	77.38 (1.04)	67.00 (0.90)	45.38	35.00	2.42	2.09	141.81	109.38
Cyclophosphamide (Alkylating agent)	Inj. 500 mg/ vial	35.00 (0.47)	72.20 (0.97)	59.50 (0.80)	37.20	24.50	2.06	1.70	106.29	70.00
Dacarbazine (Alkylating agent)	Inj. 200 mg / vial	188.00 (2.53)	476.19 (6.40)	360.00 (4.84)	288.19	172.00	2.53	1.91	153.29	91.49
Daunorubicin HCl Antibiotic	Inj. 20 mg/ vial	204.00 (2.74)	398.62 (5.36)	371.00 (4.99)	194.62	167.00	1.95	1.82	95.40	81.86
Docetaxel (Plant alkaloids; antimicrotubule agent)	Inj. 120 mg/ 3 ml	2700.00 (36.30)	18700.00 (251.38)	14916.00 (200.51)	16000.00	12216.00	6.93	5.52	592.59	452.44
	Inj. 80 mg/ 2 ml	1800.00 (24.20)	11760.80 (158.10)	10550.00 (141.82)	9960.80	8750.00	6.53	5.86	553.38	486.11
Doxorubicin (Antibiotic, inhibits topoisomerase II activity)	Inj. 10 mg/ vial	76.49 (1.03)	254.00 (3.41)	128.86 (1.73)	177.51	52.37	3.32	1.68	232.07	68.47
	Inj. 50 mg/ 25 ml vial	250.47 (3.37)	8585.00 (115.41)	2555.00 (34.35)	8334.53	2304.53	34.28	10.20	3327.56	920.08
Etoposide (Plant alkaloids; topoisomerase II inhibitors)	Inj. 100 mg in 5 ml	76.00 (1.02)	196.00 (2.63)	182.00 (2.45)	120.00	106.00	2.58	2.39	157.89	139.47
Gemcitabine (Anti-metabolite)	Inj. 1000 mg/ vial	836.00 (11.24)	6265.00 (84.22)	3700.00 (49.74)	5429.00	2864.00	7.49	4.43	649.40	342.58
	Inj. 200 mg/ vial	240.00 (3.23)	1495.00 (20.10)	865.37 (11.63)	1255	625.37	6.23	3.61	522.92	260.57
Hydroxyurea (antimetabolite)	Tab. 500 mg 10's	6.00 (0.08)	12.631 (0.17)	12.20 (0.16)	6.63	6.20	2.11	2.03	110.52	103.33
Imatinib mesylate (signal transduction inhibitor,Protein-tyrosine kinase inhibitor)	Tab. 400 mg 10's	35.00 (0.47)	213.30 (2.87)	210.00 (2.82)	178.30	175.00	6.09	6.00	509.43	500.00
Methotrexate (Antimetabolite and antifolate agent)	Tab. 2.5 mg/ 10's	4.00 (0.05)	5.60 (0.08)	1.89 (0.03)	1.60	-2.11	1.40	0.47	40.00	-52.85
Oxaliplatin (Alkylating-agent)	Inj. 50 mg/ vial	475.00 (6.39)	3425.00 (46.04)	2250.00 (30.25)	2950.00	1775.00	7.21	4.74	621.05	373.68
Paclitaxel (Plant alkaloids; Antimicrotubule agent)	Inj. 100 mg/ 16.7 ml	540.00 (7.26)	4085.00 (54.91)	3452.00 (46.40)	3545.00	2912.00	7.56	6.39	656.48	539.26
Temozolomide (alkylating agent)	Tab. 250 mg (5 tab/ bottle)	537.00 (7.22)	5088.50 (68.40)	3120.00 (41.94)	4551.50	2583.00	9.48	5.81	847.58	481.01
	Tab. 100mg (5 tab/ bottle)	292.60 (3.93)	2134.00 (28.69)	994.00 (13.36)	1841.40	701.40	7.29	3.40	629.32	239.71
Vincristine (Plant alkaloids; Mitotic inhibitors)	Inj. 1 mg/ ml vial	25.00 (0.34)	156.00 (2.10)	52.00 (0.70)	131.00	27.00	6.24	2.08	524.00	108.00
Hormone modulators										
Anastrozole (nonsteroidal aromatase inhibitor)	Tab. 1 mg. 10's	7.80 (0.10)	57.64 (0.77)	55.00 (0.74)	49.84	47.20	7.39	7.05	638.97	605.13
Bicalutamide (antiandrogen)	Tab. 50 mg 10's	13.70 (0.18)	57.06 (0.77)	51.2 (0.69)	43.36	37.50	4.16	3.74	316.5	273.72
Letrozole (aromatase inhibitors)	Tab. 2.5 mg 10's	3.40 (0.05)	38.80 (0.52)	6.00 (0.08)	35.4	2.60	11.41	1.76	1041.18	76.47
Leuprolide Acetate (Gonadotropin-releasing hormone receptor agonist)	Inj. 3.75 mg/ vial	1988.00 (26.72)	9250.00 (124.34)	4200.00 (56.46)	7262.00	2212.00	4.65	2.11	365.29	111.27
Tamoxifen Citrate (nonsteroidal selective estrogen receptor modulator)	Tab. 10 mg 10's	1.00 (0.01)	3.80 (0.05)	1.82 (0.02)	2.80	0.82	3.80	1.82	280.00	82.40
Modulating immune system										
Bortezomib (Immunotherapeutic drug)	Inj. 3.5 mg	3188.00 (42.86)	18988.00 (255.25)	7571.00 (101.77)	15800.00	4383.00	5.96	2.37	495.61	137.48
Lenalidomide (Immunomodulatory agents)	Tab. 10 mg 10's	46.40 (0.62)	290.00 (3.90)	76.90 (1.03)	243.6	30.5	6.25	1.66	525.00	65.73
	Tab. 20 mg 10's	1.60 (0.02)	4.90 (0.07)	2.50 (0.03)	3.30	0.90	3.06	1.56	206.25	56.25
Bisphosphonate										
Zoledronic acid (bisphosphonate inhibits osteoclast function and prevents bone resorption)	Inj. 4 mg/ vial	165.00 (2.22)	3212.00 (43.18)	1357.47 (18.25)	3047.00	1192.47	19.47	8.23	1846.67	722.71

The cost of methotrexate tablet in the cheapest branded drug was lesser than the Jan Aushadhi (INR 1.89 (cheapest brand) vs. INR 4.00 (Jan Aushadhi generic drug)), while the costly branded was higher (INR 4.00 (Jan Aushadhi generic drug) vs. INR 5.60 (costliest brand)). The highest CD with regard to Indian rupees was seen for the cytotoxic drug docetaxel injection (Jan Aushadhi INR 2,700 vs. costliest branded INR 18,700 vs. cheapest branded INR 14,916)) while the least was observed for methotrexate 2.5 mg tablet (Jan Aushadhi INR 4.00 vs. costly branded INR 5.60 vs. cheap branded INR 1.89)).

The CR analysis results showed 1.40 to 34.28 fold compared to the costliest brand and 0.47 to 10.20 fold compared to the cheapest branded drug. Compared with the costliest drug, the highest CR of 34.28 was observed for doxorubicin (50 mg injection vial) and the lowest of 1.40 for methotrexate (2.5 mg tablet 10s). However, compared with the cheapest drug, the highest CR of 10.20 was observed again for doxorubicin (50 mg injection vial) and the lowest of 0.47 for methotrexate (2.5 mg tablet 10s). The methotrexate tablet from the cheapest branded drug was INR 1.89 as against INR 4.00 for the Jan Aushadhi generic drug. The results of the CV also showed a 3327.56 (doxorubicin; 50 mg injection vial) to 40 (methotrexate; 2.5 mg tablet 10's) when Jan Aushadhi generic drugs was compared to the costliest branded drugs, and 920.08 (doxorubicin; 50 mg injection vial) to -52.85 (methotrexate; 2.5 mg tablet 10s) when the Jan Aushadhi drugs were compared to the cheapest branded drugs.

## Discussion

The costs of cancer drugs can be exorbitant for most families. The resulting financial toxicity can be very severe on the socioeconomically marginalized, impoverished people, and the elderly depending on their pension or saved income [19]. The study results clearly show that except for methotrexate (2.5 mg tablet), the cost of anticancer branded drugs was more than their generic counterparts sold through Jan Aushadhi pharmacies. This is the first cost comparison study that addresses all the anticancer drugs available at Jan Aushadhi Kendra compared with the branded drugs marketed in India. Our results substantiate the earlier observations of Kashyap and co-workers [13]. They have also reported that replacing generic anticancer drugs can result in substantial cost savings and benefit the patient requiring curative chemotherapy for breast cancer.

The key observation of this study is that the cost of Jan Aushadhi generic drugs were significantly less in the case of the most important cytotoxic drugs like paclitaxel, docetaxel, doxorubicin, carboplatin, cisplatin, gemcitabine, oxaliplatin, capecitabine, and etoposide that are used either alone or in combination to treat different cancers. Of these, low doses of cisplatin or carboplatin are used with radiation (chemo-irradiation) to treat head and neck, oesophageal, and cervical cancer. In contrast, oxaliplatin and capecitabine are used for rectal cancers to maximize the radiation cell kill to achieve complete remission and disease-free survival [20,21]. In addition to this, zoledronic acid, a bisphosphonate that inhibits bone resorption, is used in conjunction with standard antineoplastic therapy to mitigate cancer-related hypercalcemia and for people affected with multiple myeloma and metastasis from solid tumors to reduce skeletal-related complications. It has proved immensely useful in improving the quality of life of the affected individual [22].

A drug audit suggests that, in India, almost 80% of all drugs are marketed as branded molecules and are more expensive than their unbranded generic counterparts [23]. However, several studies have shown that generic drugs are as effective as branded drugs and compliant with the Indian Pharmacopoeia standards for quality [24]. Additionally, comparative studies have also affirmed that substitution with generics can result in almost 15% savings on the medication cost [25]. When considering the total healthcare expenditure, the cost of medicines and pharmaceutical products forms a major part, and deliberate attempts are being made at reducing the burden to the patient [7,10]. In this regard, reports from Europe and developing countries have shown that generic drugs can reduce the medicine bill substantially [25].

Globally, the cost of cancer drugs varies in different countries, and this disparity depends on the individual and family's national and personal wealth [8]. However, of all countries, the cost of anticancer drugs is reported to be highest in the United States [8,26], and reports also suggest that when compared to the general public, an individual diagnosed with cancer is at 2.7 times risk of declaring bankruptcy [27]. In addition, the financial toxicity of the drugs compels the patient/their family members to refuse treatment and/or discontinue the prescribed therapy [8,28]. Similar observations have also been reported from Japan, where studies have shown that most cancer patients experience a significant financial burden of cancer treatment and that approximately 5.7% of them declined medical care due to cost factors [29].

Multiple reports from India have clearly shown that out-of-pocket expenditure for cancer treatment is highest for any ailment. The amount in private hospitals can be as high as three times spent in public/government hospital facilities [2]. The treatment costs are exorbitant, and more than 60% of the people treated in the private sector expend nearly 20% of their annual per capita household expenditure for cancer care of the affected family member [2]. Reports also suggest that in India, almost 40% of people requiring cancer admission seek financial assistance from friends, relatives, or donor organizations or by using savings or the sale of assets [2]. Recent reports also suggest that compared to Australia, China, Israel, South Africa, the United Kingdom, and the United States, the cost of affordability analysis was high for India [8]. When considered in total, all these observations affirm that the financial toxicity is enormous on an Indian family that has to care for a member affected with cancer, and methods to mitigate the burden are a necessity.

## Conclusions

For the first time, our study presents the comparative CD between Jan Aushadhi generic drugs with the costliest and cheapest branded drugs used in cancer care in India. From a social perspective, decreasing the cost of drugs will improve affordability, medication compliance, and the patient's financial burden. Therefore, the need of the hour is to make the treating physicians aware of the comparative difference in cost of these drugs, their financial benefit to the marginalized patients, and encourage the prescription of Jan Aushadhi medicines. In addition to this, the general public should also be made aware of the benefit of the Jan Aushadhi scheme. Endeavors in these directions will help popularize Jan Aushadhi generic drugs and benefit the marginalized people of society.
